# Zinc transporter ZIP7 is a novel determinant of ferroptosis

**DOI:** 10.1038/s41419-021-03482-5

**Published:** 2021-02-19

**Authors:** Po-Han Chen, Jianli Wu, Yitong Xu, Chien-Kuang Cornelia Ding, Alexander A. Mestre, Chao-Chieh Lin, Wen-Hsuan Yang, Jen-Tsan Chi

**Affiliations:** 1 Department of Molecular Genetics and Microbiology, Duke University Medical Center, Durham, NC 27708 USA; 2 Duke Center for Genomic and Computational Biology, Duke University, Durham, NC 27708 USA; 3 Department of Biochemistry, Duke University Medical Center, Durham, NC 27708 USA

**Keywords:** Necroptosis, Transcriptomics, Apoptosis

## Abstract

Ferroptosis is a newly described form of regulated cell death triggered by oxidative stresses and characterized by extensive lipid peroxidation and membrane damages. The name of ferroptosis indicates that the ferroptotic death process depends on iron, but not other metals, as one of its canonical features. Here, we reported that zinc is also essential for ferroptosis in breast and renal cancer cells. Zinc chelator suppressed ferroptosis, and zinc addition promoted ferroptosis, even during iron chelation. By interrogating zinc-related genes in a genome-wide RNAi screen of ferroptosis, we identified *SLC39A7*, encoding ZIP7 that controls zinc transport from endoplasmic reticulum (ER) to cytosol, as a novel genetic determinant of ferroptosis. Genetic and chemical inhibition of the ZIP7 protected cells against ferroptosis, and the ferroptosis protection upon ZIP7 knockdown can be abolished by zinc supplementation. We found that the genetic and chemical inhibition of ZIP7 triggered ER stresses, including the induction of the expression of *HERPUD1* and *ATF3*. Importantly, the knockdown of *HERPUD1* abolished the ferroptosis protection phenotypes of ZIP7 inhibition. Together, we have uncovered an unexpected role of ZIP7 in ferroptosis by maintaining ER homeostasis. These findings may have therapeutic implications for human diseases involving ferroptosis and zinc dysregulations.

## Introduction

Ferroptosis is a novel form of regulated cell death^1,2^ with distinct morphological, genetic, and biochemical features. Ferroptosis was first described as a death mechanism by which erastin-induced cell death^3^. Erastin was found to be an inhibitor of xCT (encoded by *SLC7A11)*, a cystine importer in exchange for glutamate export. Therefore, erastin blocked cystine import, deplete intracellular glutathione, resulting in the accumulation of lipid-based reactive oxygen species (ROS), membrane damage, and ferroptotic death. The lipid peroxidation can be neutralized by two different mechanisms of ferroptosis protection, either GPX4 (glutathione peroxidase 4) or ferroptosis suppressor protein 1 (FSP1). GPX4 is a phospholipid hydroperoxidase that utilizes GSH as a co-factor to neutralize ROS^2^. Therefore, ferroptosis can also be triggered by either the depletion of GSH or direct inhibition of GPX4. Reciprocally, the activation of NRF2 and GSH generation can robustly protect cells from ferroptosis^4,5^. Recently, FSP1 is discovered as a new ferroptosis protection mechanism via CoQ_10_ as a lipophilic radical-trapping antioxidant^6,7^.

As the name implies, one canonical feature of “ferroptosis” is the iron-dependency. The iron chelator blocked ferroptosis and the addition of iron sensitized cells to ferroptosis. The hemochromatosis hepatocytes^8^ and erythrocyte-ingested macrophages^9^, with elevated iron levels, are highly susceptible to ferroptosis. Furthermore, many genetic determinants of ferroptosis regulate ferroptosis by affecting iron metabolism^10–12^. Indeed, a recent report has identified the transferrin receptor as a biomarker of ferroptosis^13^. While the role of iron in ferroptosis is well recognized, much remains unknown regarding the underlying mechanisms. Iron is postulated to promote lipid peroxidation and ferroptosis via the non-enzymatic Fenton reaction that amplifies ROS^14^.

Other than iron, it is not clear whether any other transition metals also regulate ferroptosis. In the original ferroptosis study that revealed the essential role of iron^1^, several different metals, including manganese, nickel, cobalt, or copper, were also tested together with iron, but their effects on ferroptosis were limited. Here, we show that ferroptosis sensitivity is also significantly affected by zinc, a divalent metal ion crucial for many biological processes^15^. We found that zinc chelator protected ferroptosis, and zinc addition promoted ferroptosis. Since zinc itself cannot move across the membrane, its movement among different cell compartments is controlled by two classes of transporters: the SLC39 family (ZIP, Zrt-like, and Irt-like protein family members) and SLC30 (ZNT, zinc transporter) family^16^. SLC39 family members transport zinc into the cytosol from either the extracellular space or intracellular stores such as the endoplasmic reticulum (ER). In contrast, the SLC30 family members mediate zinc efflux from the cytosol to other cellular compartments^17^. Among these zinc transporters, we found ZIP7, a member of the *SLC39* transporter that promotes cytosolic zinc levels, was essential for ferroptosis. The genetic knockdown and chemical inhibition of ZIP7^18^ conferred robust ferroptosis protection. Mechanically, ZIP7 inhibition triggers the ER stress response, especially the induction of HERPUD1, which contributes to ferroptosis protection. Together, these data revealed the unexpected role of zinc and ZIP7 in regulating ferroptosis via organellar communication between ER and nuclei.

## Results

### Manipulations of zinc affected ferroptosis sensitivity

**Fig. 1:**
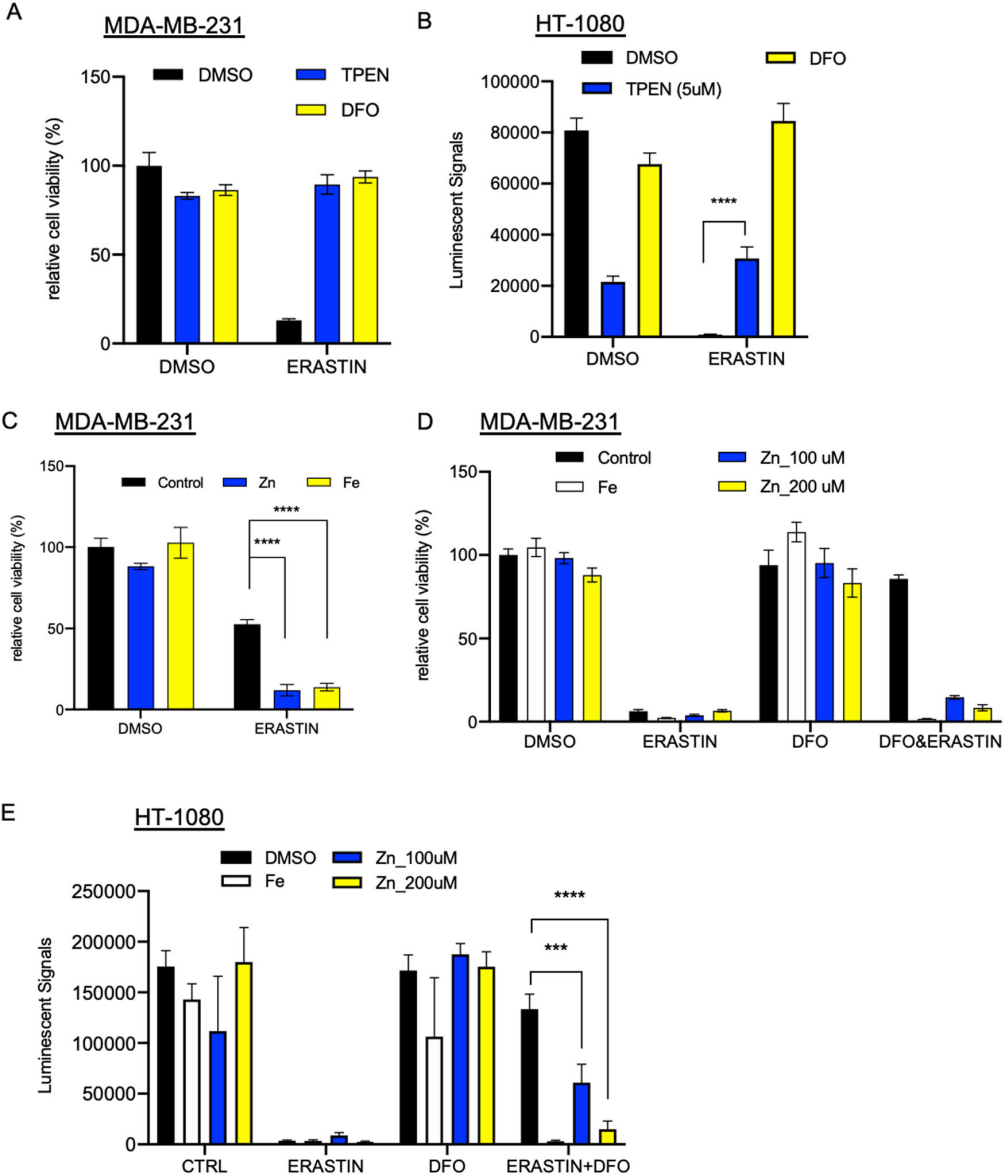
Zinc treatment affects ferroptosis susceptibility. **A** MDA-MB-231 cells were treated with DMSO, 5 μM TPEN (zinc chelator), 100 μM DFO (iron chelator) together with 10 μM erastin for 21 h. **B** HT-1080 cells were treated with DMSO, 5 μM TPEN (zinc chelator), 100 μM DFO (iron chelator) together with 10 μM erastin for 24 h. The data were represented as mean ± SD (*n* = 6; ****p < 0.0001; two-way ANOVA) (**C**) MDA-MB-231 cells were treated with 4 μM erastin alone or combined with 100 μM ZnCl_2_ or 25 μM Ferric Citrate for 24 h. The data were represented as mean ± SD (*n* = 3; ****p < 0.0001; two-way ANOVA). **D** Zinc addition mitigated the ferroptosis protective effects of an iron chelator. MDA-MB-231 cells were treated with 5 μM DFO, 25 μM ferric citrate, 100 or 200 μM ZnCl_2_ 10 μM erastin for 24 h. **E** HT-1080 cells were treated with 5 μM DFO, 25 μM ferric citrate, 100 or 200 μM ZnCl_2_, and 10 μM erastin for 24 hours. The data were represented as mean ± SD (*n* = 6; ***p = 0.0001; ****p < 0.0001; two-way ANOVA).

Ferroptosis is a newly recognized form of regulated cell death. As the name of ferroptosis indicates, this form of regulated cell death is an iron-dependent process. Therefore, ferroptosis can be blocked by iron chelators, such as deferoxamine (DFO). Also, excessive iron in cells promotes ferroptosis. In the original ferroptosis study^1^, iron was tested together with manganese, nickel, cobalt, or copper. Only iron was shown to affected erastin sensitivity. However, the role of zinc was not examined. To determine the potential of zinc in regulating ferroptosis, we used erastin to induce ferroptosis in two ferroptosis-sensitive cell lines MDA-MB-231 and HT-1080. We then treated them with the chelators of either iron (DFO) or zinc (N,N,N′,N′-Tetrakis(2-pyridylmethyl)ethylenediamine, TPEN). As expected, DFO significantly rescued erastin-induced ferroptosis (Fig. 1A). Surprisingly, zinc chelator TPEN also protected cells from erastin-induced death in MDA-MB-231 cells (Fig. 1A). Although HT-1080 was sensitive to TPEN under normal conditions, TPEN also significantly rescued erastin-mediated cell death (Fig. 1B). Conversely, the addition of zinc chloride (ZnCl_2_), similar to ferric citrate (FC), significantly sensitized cells to ferroptosis (Fig. 1C). This observation is consistent with a recent paper showing zinc toxicity killed A549 lung cancer cells via ferroptosis^19^.

Next, we explored the relationship between iron and zinc metabolisms. We first blocked the erastin-induced ferroptosis by DFO, followed by the addition of either FC or ZnCl_2_. As expected, FC overcame the ferroptosis protection of DFO (Fig. 1D, E). Unexpectedly, ZnCl_2_

overcame the ferroptosis protection of DFO and re-sensitized cells to ferroptosis in both MDA-MB-231 (Fig. 1D) and HT-1080 (Fig. 1E). Therefore, the zinc levels significantly affect ferroptosis’s sensitivity, a cell death mechanism intimately associated with iron-dependency.

### Genome-wide RNAi screen reveals novel genetic determinants of ferroptosis

**Fig. 2:**
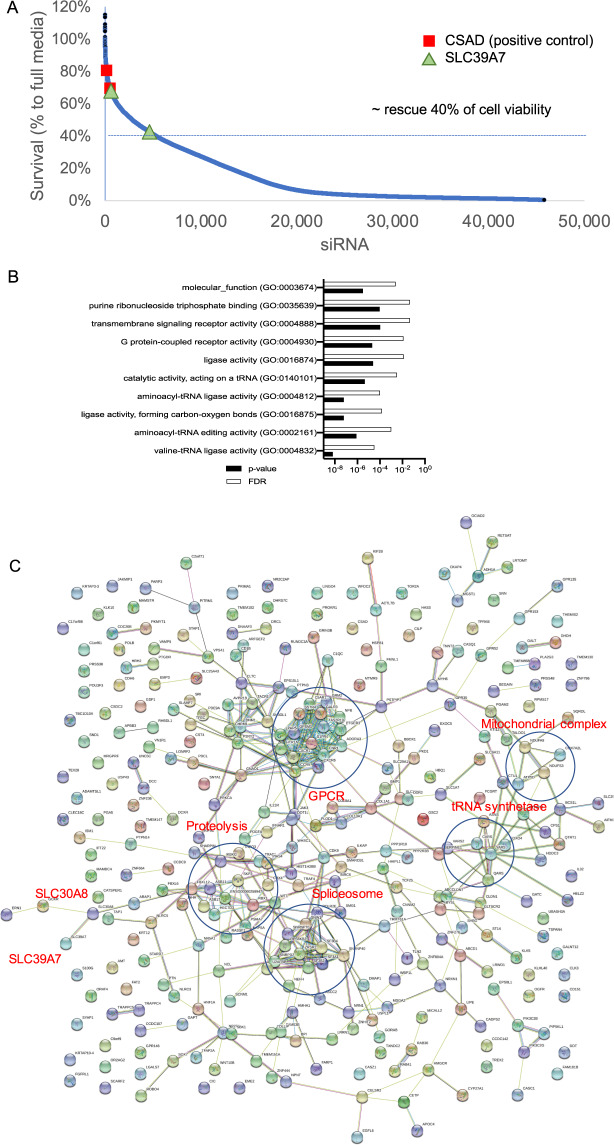
Genome-wide RNAi screens of cystine-deprivation mediated ferroptosis. **A** RCC4 cells were transfected with individual siRNA for 72 h. Cystine deprived medium (2 µM, 1% of regular media) was then applied for an additional 24 h before cell viability analysis. Cell viability is normalized to the viability in a regular medium with non-targeting siRNA on each microplate, then plotted against the rank of the viability of each siRNAs. CSAD is a positive control as shown in red squares. SLC39A7 (ZIP7) is shown in green triangles. **B**, **C** Gene Ontology (**B**), and String (**C**) analysis of 388 candidates were found to essential for ferroptosis on the screen. (FDR: false discovery rate, *P* < 0.05; *p*-value test type: Fisher’s exact).

To identify the genetic elements and biological processes involved in the ferroptosis triggered by cystine deprivation, we performed a genome-wide siRNA screen using Qiagen Human whole-genome siRNA library v1.0 covered more than 22,000 genes in the human genome (Fig. 2A). There are at least four siRNAs for each target gene, of which two siRNAs are combined into two independent pools. The siRNAs were transfected to RCC4 for 72 h, and ferroptosis was triggered by cystine deprivation. Then, each treatment’s relative cell viability was measured by ATP content (CellTiterGlo) and normalized to cells grown under full media. The gene was identified as a putative hit when both RNAi pools mitigated the cell death to at least 40% viability. These criteria identified 388 genes as essential for ferroptosis (supplemental information, Table S1).

The completed siRNA identified several genes previously known to be essential for ferroptosis. One top hit is *CSAD* (Cysteine Sulfinic Acid Decarboxylase), which mediates the limiting steps of taurine synthesis from cysteine. As previously shown^20^, the knockdown of CSAD could block the taurine synthesis to preserve the cysteine for GSH synthesis to neutralize the ROS and rescue ferroptosis. In addition, another top hit is *CARS*, which encodes the cysteinyl-tRNA synthetase. CARS was reported to be essential for ferroptosis since its depletion enhanced the transsulfuration pathway^20^. The genomic screen also identified several genes necessary for ferroptosis, including *MESH1* (the first cytosolic NADPH phosphatase^21^) and relevant TAZ-regulated target genes^22,23^. From the top identified hits, several processes were enriched based on Gene Ontology (GO) analysis^24^, including amino acid metabolism processes, ubiquitin-dependent proteasome activity, mitochondrial complex I, and vacuole ATPase activity (Fig. 2B). String analysis^25^ of protein-protein interactions identified the pathways of GPCR (G-protein coupled receptor) signaling, spliceosome, proteolysis, and tRNA synthetases (Fig. 2C). Some of these biological processes uncovered in our screens were also found to be essential for ferroptosis by other genome-wide screens^20,26^.

### The identification of zinc-related genes essential for ferroptosis

From the completed screens, we wish to identify zinc-related genes essential for ferroptosis. As mentioned above, zinc movement between different cell compartments is controlled by transporters in either the SLC39 family (ZIP) or SLC30 (ZNT) family. When all members of the ZnT/SLC30 and ZIP/SLC39A family were examined in the RNAi screening data, we found siRNA pools targeting ZIP7 (*SLC39A7*) and ZNT8 (*SLC30A8*) conferred robust ferroptosis protection (Fig. 2C). However, ZNT8 is known to be expressed mainly in the insulin-secreting β cells^27^ and not robustly expressed in most carcinoma cancer cells. Therefore, we focus on the ZIP7 as a potential zinc-related regulator of ferroptosis for further investigation.

### The genetic and chemical inhibition of ZIP7 protect cells against ferroptosis

**Fig. 3:**
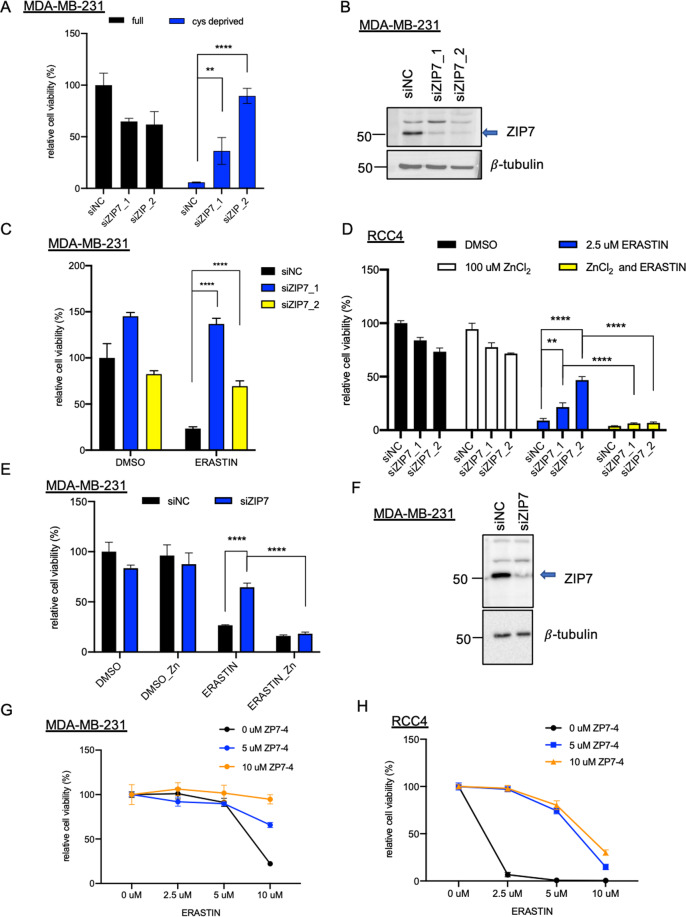
Validate the essential role of ZIP7 in ferroptosis by genetic and chemical means. **A**, **B**, **C** ZIP7 knockdown conferred resistance to cystine-deprivation or erastin mediated ferroptosis in MDA-MB-231 cells. **A** MDA-MB-231 cells were transfected with two independent siRNAs targeting ZIP7 for 48 h and treated with the cystine-deficient medium. **B** The knockdown efficiency of both ZIP7 siRNA in MDA-MB-231 was validated by the lower levels of ZIP7 protein (arrow) using Western blots. **C** MDA-MB-231 cells were transfected with indicated siRNA for 72 h and treated with 5 μM erastin for an additional 22 h before measuring the viability with CellTiter-Glo®. **D**, **E**, **F** ZIP7 knockdown conferred resistance to erastin-mediated ferroptosis in a zinc-dependent manner. **D** RCC4 cells were treated with non-targeting (NC) or individual ZIP7 siRNA for 48 h, followed by treatment of 100 μM ZnCl2, 2.5 μM erastin, or combination for an additional 24 h. **E** MDA-MB-231 cells were treated with siNC and ZIP7-targeting siRNAs for 50 h, followed by treatment of 100 μM ZnCl2, 10 μM erastin, or combination for 18 h. **F** The knockdown efficiency of ZIP7 siRNA in MDA-MB-231 was validated by Western blots (arrow for ZIP7). **G**, **H** ZIP7 inhibitor protected against erastin-mediated ferroptosis. MDAMB231 or RCC4 cells were pretreated with ZIP7 inhibitor (NVS-ZP7-4 (48 h for 231 and 24 h for RCC4) followed by the addition of erastin for an additional 24 h. The cell viability was measured by CellTiter-Glo®. The data were represented as mean ± SD (*n* = 3; **p < 0.01; ****p < 0.0001; two-way ANOVA).

Next, we wished to validate that ZIP7 was essential for ferroptosis. First, we found that ZIP7 knockdown by additional independent sets of siRNAs significantly rescued the ferroptosis of MDA-MB-231 cells induced by cystine deprivation (Fig. 3A, B) or erastin (Fig. 3C). Furthermore, the ferroptosis protection effects of the ZIP7 knockdown can be abolished by zinc supplementation in RCC4 (Fig 3D) and MDA-MB231 (Fig. 3E, F). This result was also validated by the CellTox-Green assay, which measured cell death based on the released cellular DNA (Fig S1A). Besides RCC4 and MDA-MB-231, we also observed ZIP7/zinc-dependency in other ferroptosis sensitive cells, such as HT-1080 (Fig S1B). Together, ZIP7 knockdown rescued ferroptosis of MDA-MB-231, RCC4, and HT-1080. Furthermore, ZnCl_2_ treatment abolished ferroptosis protection by ZIP7 knockdown.

A recent study identified a potent and specific ZIP7 inhibitor NVS-ZP7-4^18^. To determine whether the chemical inhibition of ZIP7 also protected ferroptosis, we treated the MDA-MB-231 with NVS-ZP7-4 together with erastin. We found that NVS-ZP7-4, similar to ZIP7 siRNAs, significantly reduced the erastin-induced death of MDA-MB-231 (Fig. 3G). Similar ferroptosis rescuing effects of NVS-ZP7-4 were also seen for RCC4 (Fig. 3H). Therefore, the inhibition of ZIP7 by both genetic and chemical means provided significant protection against ferroptosis. Collectively, these data indicate the critical role of ZIP7 and zinc transport for ferroptotic cell death.

### Transcriptome response to the ZIP7 knockdown

**Fig. 4:**
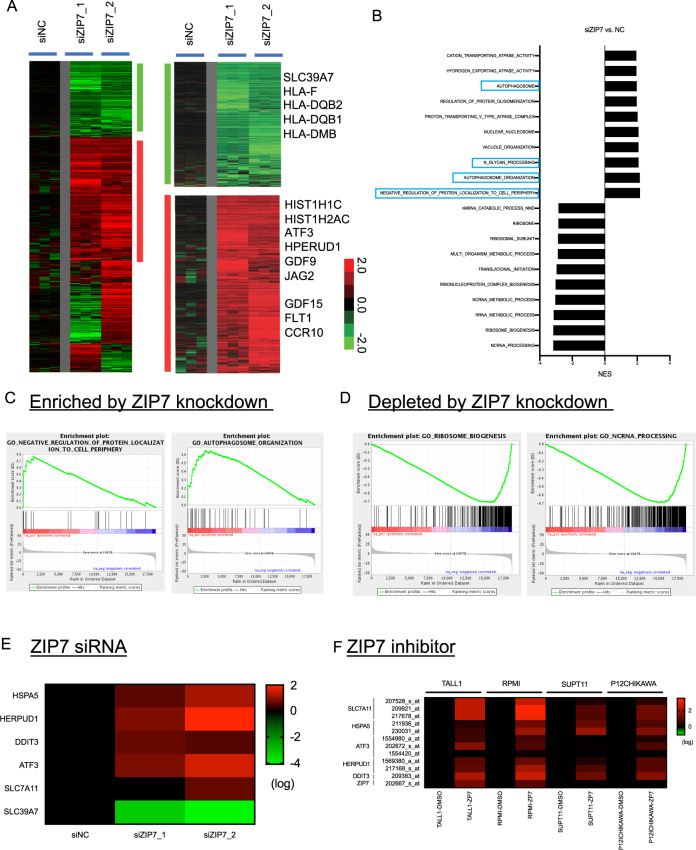
Transcriptome response to ZIP7 knockdown. **A** Heatmap of the transcriptional response to the ZIP7 knockdown. The names of selected induced (red) and repressed (green) were indicated. **B**–**D** GSEA analysis of the indicated GO: gene ontology (**B**) and showed gene-sets enriched (**C**) or depleted (**D**) by ZIP7 knockdown (*p* < 0.001)). **E**–**F** Heatmaps of the changes in the genes in the ER stress pathway in response to the treatments of either two siZIP7 (**E**) or ZIP7 inhibitor (**F**).

We hypothesized that the depletion of ZIP7 might protect ferroptosis by affecting gene expression. Therefore, we used RNA-Seq to profile the transcriptional response to ZIP7 knockdown when MDA-MB-231 was transfected with control or two ZIP7-targeting siRNAs in triplicates (Fig. 4A). We performed zero transformation against the average of the three control (siNC) samples, filtered the genes based on the change of at least 1.8-fold in four samples, and arranged by hierarchical clustering (Fig. 4A). We found that both ZIP7 siRNA consistently repressed 1012 genes and induced 1649 genes (Fig. 4A). The repressed genes include ZIP7 (SLC39A7) and many Major Histocompatibility Complex (MHC) genes, including HLA-F, HLA-DQB2, HLA-QB1, and HLA-DMB. The induced genes included genes involved in the endoplasmic reticulum (ER) stress (*ATF3*-Activating transcription factor 3) and *HERPUD1-*(Homocysteine Inducible ER Protein With Ubiquitin Like Domain 1), nucleosome assembly (HIST1H1C and HIST1H2AC), and cellular communications (GDF9, GDF15, FLT1, JAG2, and CCR10). The top ten enriched biological pathways identified by Gene Set Enrichment Analysis (GSEA) include the enrichment of genes involved in autophagosome, N-glycan, or protein trafficking (Fig. 4B, C), consistent with previous reports^18,28^. Besides, ZIP7 knockdown also leads to the depletion of rRNA processing, ncRNA processing, and ribosome biogenesis (Fig. 4B, D).

### ZIP7 inhibition induced ER stresses

As noted, ZIP7 knockdown induced *ATF3* and *HERPUD1* in the ER stress or unfolded protein response (UPR) (Fig. 4A)^29,30^. Therefore, we examined the effects of ZIP7 knockdown on the genes in the ER/UPR pathways. From our transcriptome analysis, ZIP7 knockdown in MDA-MB-231 significantly induced the expression of *HSPA5* (heat shock protein family A member 5, or BIP), *HERPUD1* (homocysteine induced ER protein with ubiquitin-like domain 1), *DDIT3* (DNA-damage-inducible transcript 3, or CHOP), *ATF3* (activating transcription factor 3), and *SLC7A11* (cystine/glutamate transporter) (Fig. 4E). Consistently, one independent profiling of the transcriptional response of four different cell lines to ZIP7 inhibitors also revealed the induction of *HSPA5*, *HERPUD1*, *DDIT3*, *ATF3*, and *SLC7A11* mRNA (Fig. 4F)^18^. Therefore, ZIP7 inhibition by genetic and chemical means both robustly activated ER stress expression program. This finding is consistent with previous reports of the induction of ER stresses upon ZIP7 removal in multiple biological contexts and model organisms^18,31,32^.

**Fig. 5:**
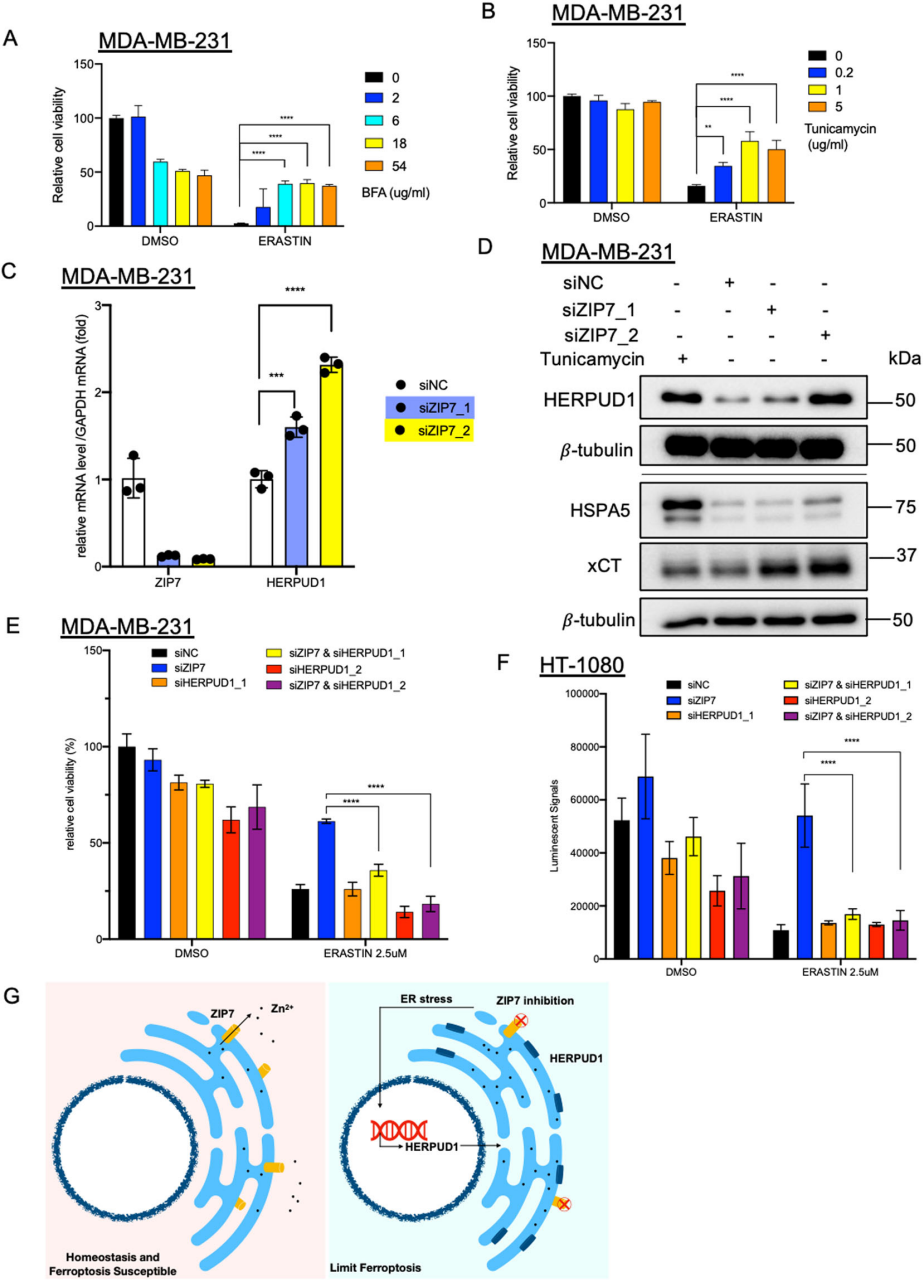
ER stress response and HERPUD1 induction confer ferroptosis resistance mediated by ZIP7 inhibition. **A**, **B** Mild induction of ER stress by chemical means protected cells against ferroptosis. MDA-MB-231 cells were pretreated with Brefeldin A (BFA, A) or tunicamycin (**B**) for 3 h, followed by 10 uM erastin for 24 h before measuring the viability with CellTiter-Glo®. The data were represented as mean ± SD (*n* = 3; ** *p* < 0.01; ****p < 0.0001; two-way ANOVA). **C** The changes in the levels of ZIP7 and HERPUD1 mRNA upon ZIP7 knockdown was validated by qRT-PCR. MDAMB231 cells were transfected with ZIP7 siRNA, and RNA samples were collected for qRT-PCR after 48 h of transfection. The data were represented as mean ± SD (*n* = 3; ***p < 0.001; ****p < 0.0001; one-way ANOVA). **D** Induction of HERPUD1 and xCT protein upon ZIP7 knockdown. Cell lysates were collected after 72 h transfection of non-targeting control (NC) or ZIP7 siRNA. For tunicamycin control, cells were treated with 0.2 μg/ml of tunicamycin for 20 h. **E**, **F** HERPUD1 induction conferred ZIP7-mediated ferroptosis resistance. MDAMB231 (**E**) or HT-1080 (**F**) cells were transfected with either siNC, or siZIP7_2, siHERPUD1, or both siRNAs for 48 h, and treated with the indicated concentration of erastin for an additional 24 h. The cell viability was measured by CellTiter-Glo®. The data were represented as mean ± SD (*n* = 3; ****p < 0.0001; two-way ANOVA). **G** Working model for ZIP7 knockdown mediated ferroptosis resistance. ZIP7 exports zinc ion into the cytosol from ER to maintain zinc homeostasis. In the absence of ZIP7, zinc accumulates in the ER, triggering ER stress response and the induction of HERPUD1 mRNA and protein expression to protect cells against ferroptosis.

Since ER stress was induced by erastin^33^ and implicated in ferroptosis^34,35^, we tested if induction of ER stress is associated with ZIP7-mediated ferroptosis protection in our systems. ER stress can be triggered by brefeldin A (BFA) or tunicamycin, both of which protected cells against ferroptosis (Fig. 5A, B). Therefore, we speculated that the induction of ER stress genes might contribute to the ferroptosis protection upon ZIP7 inhibition. Among these ER stress genes induced by ZIP7 inhibition, HSPA5 has been previously reported to protect against ferroptosis^34^. However, the knockdown of HSPA5, together with ZIP7, did not mitigate the ferroptosis protection (data not shown). Therefore, we tested the role of other ER stress genes induced by ZIP7 knockdown.

HERPUD1 is a well-established ER stress-induced gene and a potential candidate in our study since its expression is strongly induced by both siZIP7 (Fig. 4D) and ZIP7 inhibitors (Fig. 4E). First, qRT-PCR revealed that HERPUD1 mRNA was significantly upregulated upon the ZIP7 knockdown (Fig. 5C). HERPUD1 protein was also elevated by tunicamycin and by both siZIP7s siRNA (Fig. 5D). Meanwhile, we also observed induction of xCT protein, but not HSPA5, by two ZIP7 siRNAs (Fig. 5D). The lack of increased HSPA5 protein was consistent with the inability of HSPA5 to mitigate the ferroptosis protection upon the ZIP7 knockdown. Most importantly, the knockdown of HERPUD1 significantly mitigated the ferroptosis protection by ZIP7 inhibition in both MDA-MB-231 and HT-1080 (Fig. 5E, F). Therefore, the induction of *HERPUD1* contributed to the ferroptosis protection of ZIP7 inhibition. In sum, our study supports a model in which ZIP7 depletion and inhibition protect ferroptosis. The ZIP7 inhibition triggered the ER stresses, which triggered the induction of the *HERPUD1* mRNA. The translated HERPUD1 protein will then migrate back to the ER and mediate an unknown process to protect ferroptosis (Fig. 5G).

## Discussion

This study has provided compelling evidence that zinc and ZIP7 regulate ferroptosis, a regulated cell death process previously only known to be iron-dependent. Through the careful analysis of all zinc transporters in our RNAi screens, we identified ZIP7 as a novel determinant of ferroptosis. The genetic and chemical inhibition of ZIP7 robustly protects cancer cells from ferroptosis. We also found that ZIP7 knockdown may protect ferroptosis by upregulating HERPUD1, a well-known gene induced during ER stresses. Together, these data strongly indicate the unexpected role of ZIP7 in regulating ferroptosis through maintaining ER homeostasis and organellar communication.

Zinc is an essential metal required for the regulation of proliferation, metabolism, and cell signaling^36^. Zinc serves as an important intracellular second messenger^37^. Zinc deficiency leads to impaired immunity, growth retardation, poor wound healing, hair loss, diarrhea, delayed sexual maturation^38^. On the other hand, zinc toxicity may lead to nausea, vomiting, diarrhea, altered copper and iron function, and reduced immune function^38,39^. Given zinc’s importance, the levels and distribution of zinc are tightly regulated by the ZIP and ZnT family of zinc transporters, and their dysregulations lead to various pathological conditions^17,36^. ZIP7 regulates cytosolic zinc levels by allowing the transport of zinc from the ER and other organelles^40^. The importance of ZIP7 during development at the organismic levels is shown by the various phenotypic manifestation of ZIP7 deficiency. *Zip7*-deficient mice are embryonic lethal, and the hypomorphic alleles or tissue-specific removal of *Zip7* in mice blocks B cell development^41^, the dermis^32^, and death of intestinal progenitors^31^. The loss-of-function of ZIP7 homolog in *Drosophila* and zebrafish also resulted in defects in wings^42^ and neurodevelopment^43^. Therefore, ZIP7 is an evolutionarily conserved regulator critical for the proper development of multiple model organisms.

ZIP7 has been previously shown to regulate ER homeostasis, and ZIP7 removal triggered ER stress in the intestine^31^, heart^44,45^, and dermis^32^. ZIP7 removal leads to zinc accumulation in ER, which inhibits disulfide isomerase and results in protein aggregation and ER stress^32^. Consistent with these previous reports, we found that ZIP7 inhibition in cancer cells also triggered ER stress responses. Herpud1 is an ER membrane protein induced by ER stresses^46^. Herpud1 facilitates the retro-translocation of proteins from the ER to the 26 S proteasome in the cytosol for proteolytic elimination^47–49^. Interestingly, a recent study has shown that HERPUD1 protected cells from H_2_O_2_-induced cell death by regulating calcium flux by inositol 1,4,5-trisphosphate receptor (ITPR)^50^. Besides HERPUD1, other ER stress-associated genes may also contribute to ferroptosis protection. For example, xCT is expected to enhance the cystine import and GSH production to boost the ferroptosis inhibition of GPX4^51^. Therefore, the UPR-mediated induction of xCT may also contribute to the ferroptosis protection phenotypes of the ZIP7 knockdown. It is also important to point out that ZIP7 cannot fully explain the effects of zinc on ferroptosis. It has been estimated that 3000 proteins, representing ~10% of encoded mammalian proteins, interact with zinc^52^. Therefore, zinc may have much broader effects on other proteins and ferroptosis susceptibility.

Ferroptosis sensitivity is known to be associated with the various cancer cells which have developed resistance to chemotherapies or target therapeutics^23,53,54^. Interestingly, the activation of ZIP7 is indicative of tamoxifen-resistance and indispensable for the growth of resistant ER+ breast cancer^55^. Therefore, we found that activated ZIP7 is essential for ferroptosis, which may further connect the ferroptosis susceptibility to the treatment-resistant cells. Thus, ferroptosis may be used to target these ZIP7 activated tamoxifen-resistant ER+ breast tumors. The high expression/activation of ZIP7 may be used as biomarkers to identify tumor cells that are sensitive to these ferroptosis-targeting therapeutics.

Our results may have significant therapeutic implications. While triggering ferroptosis may have substantial therapeutic potential for human cancers, much remains unknown how best to select tumors that would be most responsive. Our results indicate that ZIP7 activation in tamoxifen-resistant ER+ breast tumors may be susceptible to various ferroptosis-inducing therapies. Ferroptosis is involved in many pathological conditions, such as neurodegeneration^56^, renal damages^57^, liver fibrosis^58^, cardiomyopathy^59^, and ischemia-reperfusion injuries^60^. Therefore, blocking ferroptosis using ZIP7 inhibitors may hold significant therapeutic potentials. Reciprocally, acute, and chronic zinc toxicity may result in many symptoms, including nausea, vomiting, loss of appetite, abdominal cramps, diarrhea, and headaches. Our results suggest that ferroptosis may contribute to the pathogenesis of these disease processes associated with excessive zinc.

## Materials and methods

### Cell culture and reagents

MDA-MB-231, HT-1080, and RCC4 cells were obtained from Duke Cell Culture Facility, maintained at 37 °C with 5% CO_2_, and cultured in Dulbecco’s Modified Eagle’s Medium (DMEM) with fetal bovine serum and penicillin/ streptomycin. Cell lines were authenticated by Duke CCF using short tandem repeats testing and tested mycoplasma-free. The siRNAs were obtained from the following source: ZIP7 siRNA_pool (Horizon, siGENOME SLC39A7 siRNA #M-007338-01-0005), ZIP7_1 (Horizon, #D-007338-03-0005), ZIP7_2 (Qiagen, FlexiTube siRNA, #SI04350367), HERPUD1 siRNA (Horizon, siGENOME D-020918-02-0005 (siHERPUD1_1) and D-020918-04-0005 (siHERPUD1_2)), ZnCl_2_ (Sigma, #39059), N,N,N′,N′-Tetrakis (2-pyridylmethyl) ethylenediamine (TPEN, Sigma, #P4413), ferric citrate (Sigma, #F3388), desferoxamine mesylate (DFO, Sigma, #D9533), erastin (Caymen Chemical, CAS 571203-78-6) and ZIP7 inhibitor (ZP7-4, MedChemExpress, #HY-114395 and 114395A).

### Genome-wide RNAi screen

Qiagen Human whole-genome siRNA library v1.0 was applied to clear cell Renal cell carcinoma cell line RCC4 for this screen. In the library, there are at least 4 different siRNA sequences targeting each gene, two of which are pooled together (“A + B”, “C + D”) in a well of a 384-well microplate. In each well, 1 nmole of pooled siRNAs were reverse transfected to 1000 RCC4 cells with 0.05 µl Lipofectamine RNAiMAX (ThermoFisher, 13778030) and 4.95 µl Opti-MEM (ThermoFisher, 11058021) and culture in 37 °C, 5% CO2, a humidified incubator for 72 h. Then DMEM media contains 2 µM cystine (Gibco 210130-24, supplement with L-Glutamine 4 mM, L-cystine 2 µM, and L-Methionine 30 mg/l) with 10% dialyzed fetal bovine serum (Sigma #F0392) is applied for 24 h in an incubator. Cell viability is measured by CellTiter-Glo luminescent cell viability assay (Promega, G7570) and normalized to the full media control on every plate.

### RNA extraction, primers, quantitative RT-PCR, and RNA-Seq

Total RNAs in MDA-MB-231 cells (96 h after siRNA transfection) were extracted with the RNeasy Mini Kit (QIAGEN #74104) with DNase I treatment (QIAGEN #79254). Single Read RNA sequencing was performed on the Illumina Hi-Seq 3000/4000 system at Sequencing and Genomic Technologies Shared Resource at Duke Center for Genomic and Computational Biology. The RNA-seq data were deposited into Gene Expression Omnibus (GEO) database with accession number GSE155437. To validate the differentially expressed genes uncovered from RNA-seq, cDNA was prepared using SuperScript II reverse transcriptase (Thermo Fisher Scientific #18064) with random hexamers. Quantitative RT-PCR was performed on the StepOnePlus platform (Applied Biosystems) using Power SYBR Green PCR Mix (Applied Biosystems, ThermoFisher Scientific). Primers used in the qRT-PCR were as follows: HERPUD1 (forward: 5’-TCTGGGAAGCTGTTGTTGGA; reverse: 5’-TTAGAACCAGCAGGCTCCTC), ZIP7 (forward: 5’-GGACACGCTCACAGTCATACA; reverse: 5’-CTCCTCGCCTCTTCTGAACC), and GAPDH (forward: 5’-GAGTCAACGGATTTGGTCGT; reverse: 5’-TTGATTTTGGAGGGATCTCG).

### Cell viability assay

In general, cells were pretreated (metal chelator, metal, or inhibitors) or transfected with ZIP7 siRNA for 48–72 h and treated with erastin or cystine deprivation for an additional 18–24 h as indicated in each experiment. The cell viability was determined by CellTiter-Glo (Promega), or cell death was determined by CellTox-Green (Promega) according to the manufacture’s instruction. The detailed conditions were described in the figure legends.

### Western blotting and antibodies

To evaluate the knockdown efficiency of ZIP7, cell lysates were collected by RIPA buffer 48 to 72 h after siRNA transfection. The proteins were then separated by SDS-PAGE and blotted against ZIP7 (Proteintech, #19429-1-AP), HERPUD1 (CST, #26730), HSPA5(CST, #3177), xCT (CST, #12691) or β-tubulin (Cell Signaling, CST, #2128 S) antibody as indicated. The blots were visualized by chemiluminescence and ChemiDoc Imager (Bio-Rad). The raw data for all blots are presented in supplemental Fig. 2.

### Statistical analysis

All data were presented as mean ± SD. The statistical analysis was performed by Prism 8. One-way or two-way ANOVA test was used as indicated in the figure legends.

## Supplementary information

Table s1

Figure S1 and S2
