# Supplementary material for: Zinc transporter ZIP7 is a novel determinant of ferroptosis
Source: Cell Death Dis. 2021 Feb 19;12(2):198. doi: 10.1038/s41419-021-03482-5 (PMC7895949; doi:10.1038/s41419-021-03482-5)

Figure S1 (related to Figure 3)
(A) MDA-MB-231 cells were transfected with non-targeting (NC) or ZIP7 siRNA for 48 hours, and treated with erastin (5uM), $ZnCl_2$ (100uM) or combination for 48h. The amount of cell death was indicated by fluorescent signals generated from the Cell-Tox Green reagent. (B) HT-1080 cells were treated with non-targeting (NC) or individual ZIP7 siRNA for 48 hours, followed by treatment of 100uM $ZnCl_{2,}$ 2.5uM erastin or combination for additional 24 hours. The cell viability was indicated by the luminescent signals generated from CellTiter-Glo reagents. The data were presented as mean ± SD (n=3; * p=0.0114; ****p<0.0001; two-way ANOVA)

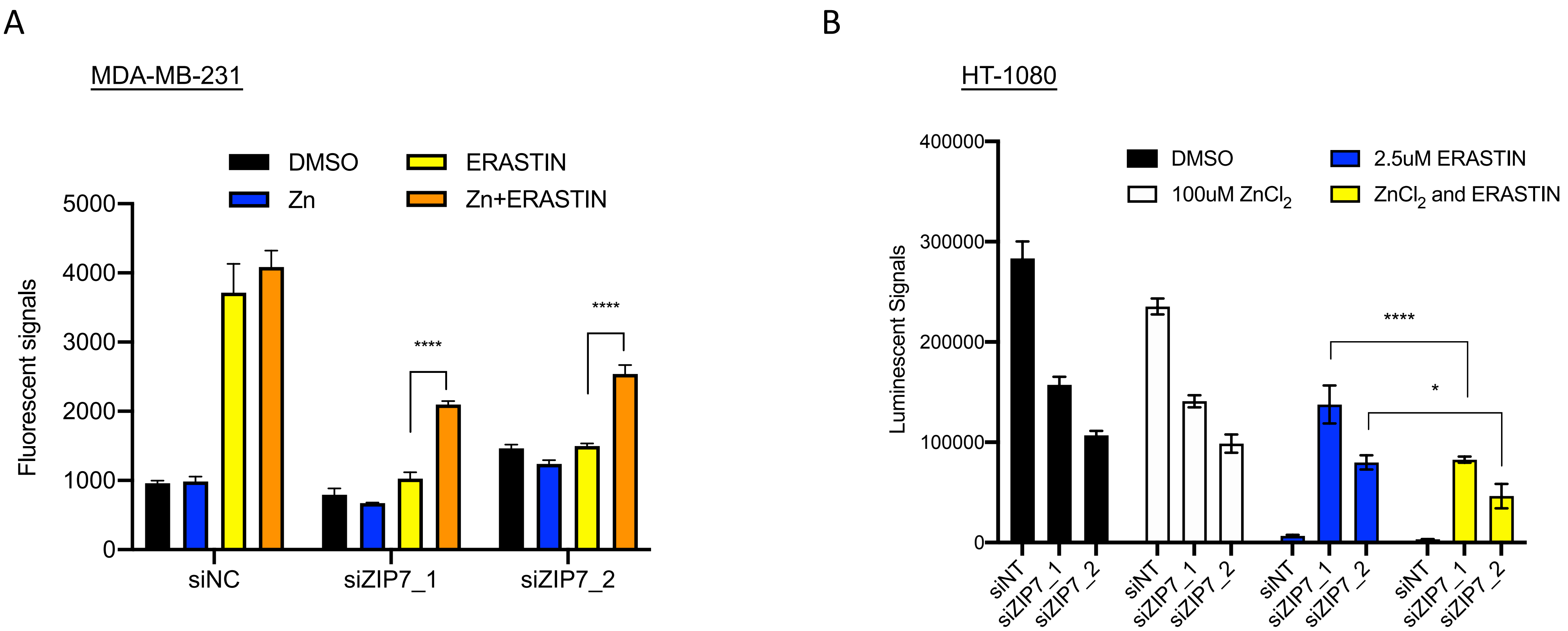

Figure S2 Original western blots for figures 3 and 5.

Related to Figure 3

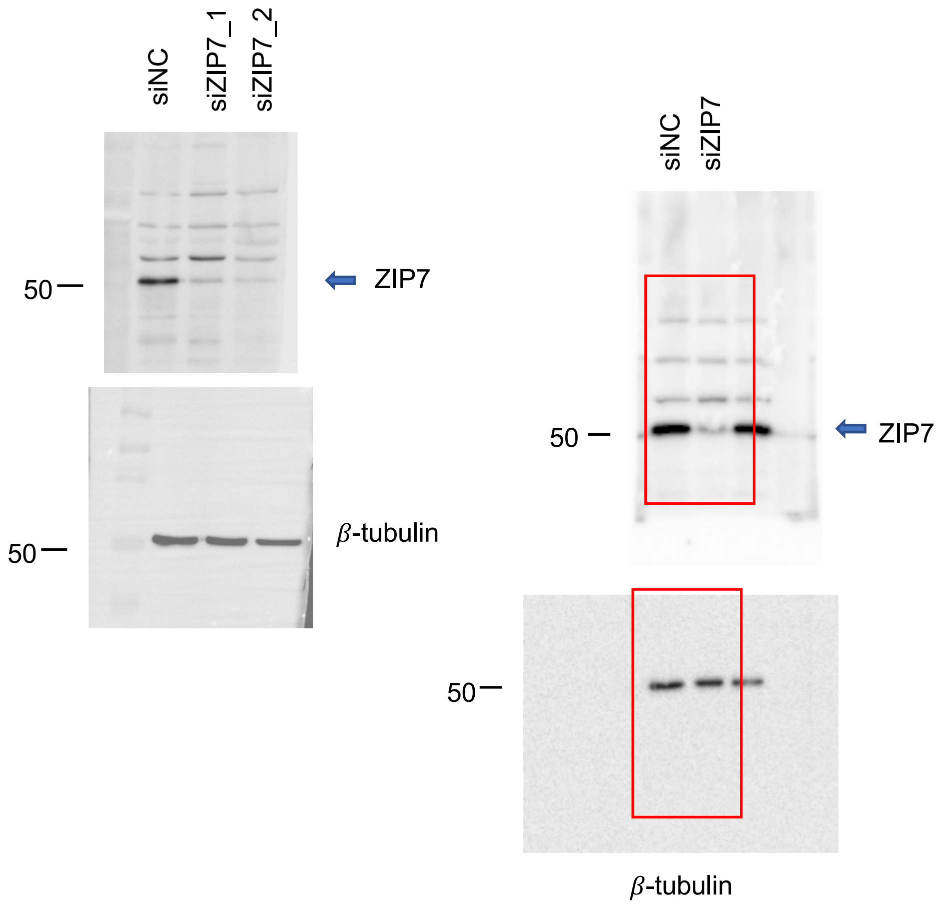

Related to Figure 5

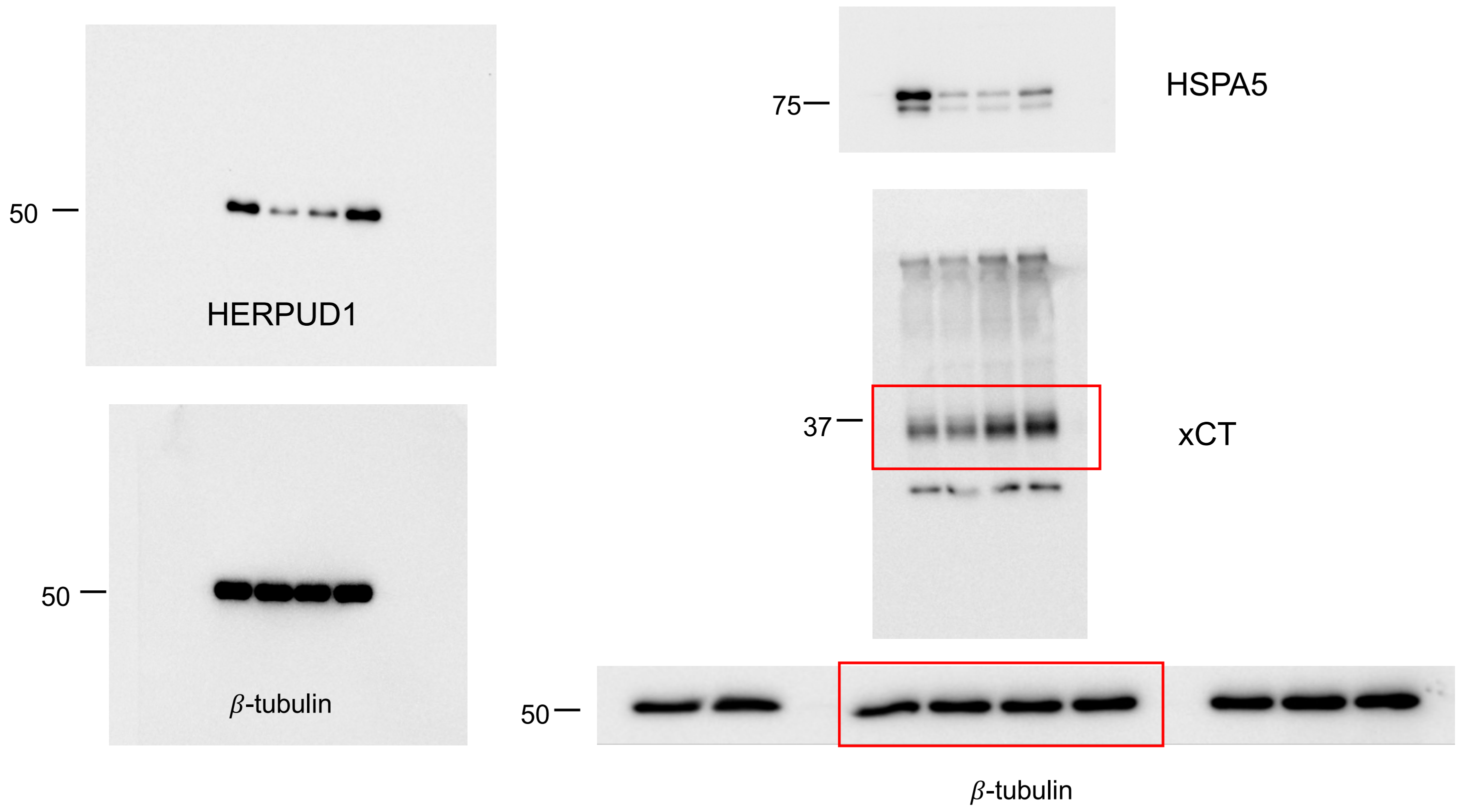

Supplement: Supplementary file 2 — Figure S1 and S2 [file 41419_2021_3482_MOESM2_ESM.pdf]
